# Does Patient Adherence Influence the Ability of Supportive Periodontal Therapy to Maintain Stability Around Teeth and Dental Implants – A Systematic Review

**DOI:** 10.3290/j.ohpd.c_2314

**Published:** 2025-10-20

**Authors:** Bernita Bush Gissler, Annika Kroeger, Gregor Würfl, Andrea Mombelli, Anton Sculean, Moritz Kebschull

**Affiliations:** a Bernita Bush Gissler Dental Hygienist and Lecturer, Department of Periodontology, School of Dental Medicine, University of Bern, Switzerland; Lecturer, University for Digital Technologies in Medicine and Dentistry, Wiltz, Luxembourg. Data curation, investigation, wrote original draft, reviewed and edited manuscript, shared first authorship with A. Kroeger.; b Annika Kroeger Clinical Lecturer and Honorary Consultant Oral Surgery, Department of Oral Surgery, School of Dentistry, Institute of Clinical Sciences, College of Medical and Dental Sciences, The University of Birmingham, Birmingham, UK. Methodology, investigation, supervision, validation, wrote original draft, reviewed and edited manuscript, shared first authorship with BB Gissler.; c Gregor Würfl Periodontologist/Implantologist, Private Practice, Wegscheid, Deutschland. Investigation, wrote and reviewed manuscript.; d Andrea Mombelli Professor Emeritus, Division of Regenerative Dental Medicine and Periodontology, University of Geneva, Geneva Switzerland. Conceptualization, validation, reviewed and edited manuscript.; e Anton Sculean Professor, Department of Periodontology, School of Dental Medicine, University of Bern, Switzerland. Conceptualization, reviewed and edited manuscript.; f Moritz Kebschull Professor, Chair of Restorative Dentistry, Periodontal Research Group, School of Dentistry, Institute of Clinical Sciences, College of Medical and Dental Sciences, The University of Birmingham, Birmingham, UK. Study design, mentor, conceptualization, methodology, supervision, reviewed and edited manuscript.

**Keywords:** adherence, compliance, periimplantitis, periodontitis, periodontal stability, supportive periodontal therapy.

## Abstract

**Purpose:**

After completion of active periodontitis and/or peri-implantitis therapy, patients transition to an individualised follow-up maintenance phase to maintain periodontal and peri-implant stability. This systematic review aims to assess whether patient adherence to supportive periodontal care(SPC) and supportive peri-implant care (SPIC) influences long-term clinical outcomes, particularly probing depth, to maintain periodontal and/or peri-implant health.

**Materials and Methods:**

Using the PECO method, the following specific question was addressed: Among successfully treated periodontitis patients with or without dental implants (P), does non-adherence (E) compared to adherence (C) to supportive periodontal or peri-implant care (SPC/SPIC) affect clinical outcomes associated with maintenance of periodontal or peri-implant health(O), based on comparative longitudinal studies (S) with a minimum follow-up of one year (T)? The following electronic databases were systematically searched: MEDLINE, Embase, Cochrane Library, Google Scholar. This was supplemented by additional manual search strategies. Due to heterogeneity in adherence definitions and outcome measures, a structured narrative synthesis was carried out (PROSPERO CRD42022371423).

**Results:**

A total of 3891 articles were selected in a primary search. Subsequently, seven studies were deemed eligible for inclusion in this review. Across these, adherence to supportive care was consistently associated with improved clinical outcomes, including reduced probing depths, lower bleeding on probing, and reduced tooth or implant loss. Due to the heterogeneity of the data, no meta-analysis was performed.

**Conclusions:**

Adherence to SPT/SPIC was consistently associated with more favorable clinical outcomes in the included studies. Future studies are necessary and should consider (i) applying consistent definitions for adherence/non-adherence based on clinical status in relation to need for intervention, (ii) time interval between SPT/SPIC intervention, (iii) consistency in data collection protocols during interventions.

After completion of the active therapy stage, patients diagnosed with periodontitis and/or peri-implantitis transition to a life-long supportive maintenance phase. Supportive periodontal therapy (SPT) and supportive peri-implant care (SPIC) aim to prevent disease recurrence through re-motivation, oral hygiene instructions, and the control of risk factors, including plaque accumulation and systemic conditions.^20,23^ The success of this phase can be defined as stable periodontal and/or peri-implant tissue without (significant) deterioration of clinical measures such as, but not limited to, pocket probing depth (probing pocket depth), bleeding on probing (BoP), and clinical attachment levels (CAL).

The topic of supportive care in periodontitis and/or peri-implantitis patients has been the centre of focus in a variety of publications, but some questions remain unresolved. It is generally accepted and acknowledged that SPT in regular intervals reduces the incidence of tooth loss over time, indicating its cruciality for long-term stability.^14,24^ According to the current guidelines,^23^ the intervals of these follow-up sessions should be scheduled according to the individual’s risk profile and periodontal status on entry of SPT. While this acknowledges the multifactorial pattern of these diseases, as well as the individual response of the affected, there is no clear guidance on or evidence of how these intervals are set. Further, only limited literature is available on adherence to SPT, reasons for non-adherence, and its effect on periodontal stability. Amerio et al^1^ noted that the lack of information as well as the lack of motivation of the patient are determinants of non-adherence.

The literature on SPIC is sparse. Carra et al^3^ investigated primordial and primary preventive strategies for peri-implantitis. A subgroup analysis included a comparison of regular attendance of patients with dental implants to non-attendance to SPIC. Meta-analysis showed a statistically significantly reduced risk of diagnosis of peri-implantitis and peri-implanti mucositis with regular SPIC on both patient and implant level.^3^ However, here too the definition of adherence, attendance, and the reasons for these are not discussed, nor were individual clinical measures assessed.

Periodontitis – being the sixth most common human disease affecting approximately 11.2% of the adult population worldwide – is a disease with a considerable global burden:^6^ It has been proposed that this oral inflammatory condition should be regarded as a non-communicable systemic disease, comparable to cardiovascular disease or diabetes mellitus.^25^ Central to the management of such diseases is regular monitoring and supportive care to maintain health, ensure long-term stability, and allow early intervention in case of deterioration.

This systematic review aims to investigate the influence of irregular attendance and/or non-adherence to SPT/SPIC on the maintenance of periodontal health by taking clinical measurements (probing pocket depth, PPD). To the best of our knowledge, this is the first study to investigate this by focussing on clinical parameters and with a special emphasis on adherence. By evaluating studies that explicitly categorized patients based on attendance behavior, the purpose of this review is to clarify whether non-adherence compromises clinical outcomes following successful treatment of periodontitis and/or peri-implantitis.

## MATERIALS AND METHODS 

This study is registered under Reg. Num: PROSPERO 2022 CRD 4202237 1423

https://.crd.york.ac.uk/prospero/display_record.php?ID=CRD42022371423

### Research Question

The following specific question constructed using the PECO method^9^ was addressed: Among successfully treated periodontitis patients with or without dental implants (P), does non-adherence (E) compared to adherence (C) to supportive periodontal or peri-implant care (SPC/SPIC) affect clinical outcomes associated with maintenance periodontal or peri-implant health (O), based on comparative longitudinal studies (S) with a minimum follow-up of one year (T)?

Population: Adults (≥18 years) with a history of successfully treated periodontitis, with or without dental implants, who are enrolled in supportive periodontal care (SPC) or supportive peri-implant care (SPIC) programs.
Exposure (E): Non-adherence to SPC/SPIC as defined by each included study.
Comparator (C): Given the heterogeneity in definitions of adherence to supportive periodontal care (SPC) and supportive peri-implant care (SPIC), we did not apply a single predefined threshold. Instead, we followed the original authors’ categorization of adherence as reported in each included study. These definitions were based on a variety of criteria, including recall intervals, attendance rates, missed appointments, or continuity of follow-up. Details of these definitions can be found in the results sections. The variability in operational definitions is acknowledged and critically appraised in the discussion section.
Outcome (O): Changes in clinical parameters reflecting periodontal or peri-implant status, including probing depth (PPD/PI-PD), bleeding on probing (BoP), tooth/implant loss, and/or other measures reflecting stability (e.g., CAL).
Study Design (S): Comparative longitudinal studies (prospective or retrospective).
Time (T): Minimum 12-month observation period following completion of active periodontal or implant therapy.

A protocol was developed and registered a priori to collect and summarize the evidence from comparative longitudinal studies (PROSPERO CRD42022371423). This manuscript was prepared according to the Preferred Reporting Items for Systematic Reviews and Meta Analysis (PRISMA) recommendations.^19^

### Search Strategy

The following electronic databases were searched systematically: MEDLINE, Embase, Cochrane Library, Google Scholar. The systematic search terms can be found in supplemental material Table S1. Additionally, a hand search of tables of content of J Clin Periodontol, J Periodontol Res, and Clin Oral Res (01-2000 – 2022) as well as experts on the topic were consulted.

Titles and abstracts identified through electronic and manual searches were independently screened by two reviewers (BB, GW). Reference lists of potentially relevant systematic reviews and selected publications were also screened for eligibility. Full texts were retrieved for articles agreed upon by both reviewers and when abstracts lacked sufficient information for decision-making. The eligibility of full texts was evaluated by two reviewers independently. Discrepancies in inclusion or exclusion decisions were resolved by consensus following consultation with a third independent reviewer (AK). An updated second search was performed prior to publication to ensure that the most recent titles were considered for inclusion.

### Inclusion Criteria

The inclusion criteria set a priori were: longitudinal comparative trials (randomized controlled trials, controlled clinical trials, observational studies: cohort studies, case-control studies), population > 20 human subjects per study group, successfully treated periodontitis patients (>18 years) with or without dental implants.

### Exclusion Criteria

The exclusion criteria set a priori were: Articles not written in English or German, as none of the authors are sufficiently fluent in other languages, unpublished works, animal trials, case reports, and consensus reports, trials with an observation period of  < 1 year, studies not reporting on our main outcome, i.e., periodontal PPD, PI-PD.

The choice of a one-year minimum follow-up period was based on pragmatic considerations. It reflects the minimum timeframe over which supportive care protocols (typically delivered at 3- to 6-month intervals) could reasonably be evaluated for their impact on clinical outcomes. This criterion aligns with thresholds used in previous systematic reviews assessing adherence in periodontal maintenance therapy.^1,14^ Importantly, all included studies exceeded this threshold, with reported follow-up durations ranging from four to twenty years. The follow-up period in each study was defined as commencing after completion of active periodontal therapy or implant placement, ensuring comparability in the maintenance phase.

### Outcome Measure

The main outcomes of interest were the changes in PPD and/or peri-implant probing depth (PI-PD) in mm between baseline and the end of the study. The measures of effect were mean changes of PPD/PI-PD in mm.

Additional outcomes include changes in BoP (%), incidence of re-instrumentation or other direct interventions, tooth or implant loss, and patient-reported outcome measures (PROMs). The measures of effect are mean changes/differences in mm, percent, and odds ratio (OR).

### Risk of Bias Assessment

The risk of bias of included articles were assessed by two independent reviewers (BB, GW) utilizing the Newcastle-Ottawa-scale (NOS) for observational studies.^26^ Disagreements between the reviewers were resolved by consensus following consultation with a third independent reviewer (AK).

### Data Extraction

Data were extracted by two independent reviewers (BB, AK). A pre-established data sheet was utilized. If data were missing, the pertinent authors were contacted.

## RESULTS

### Search Results (Fig 1)

**Fig 1:**
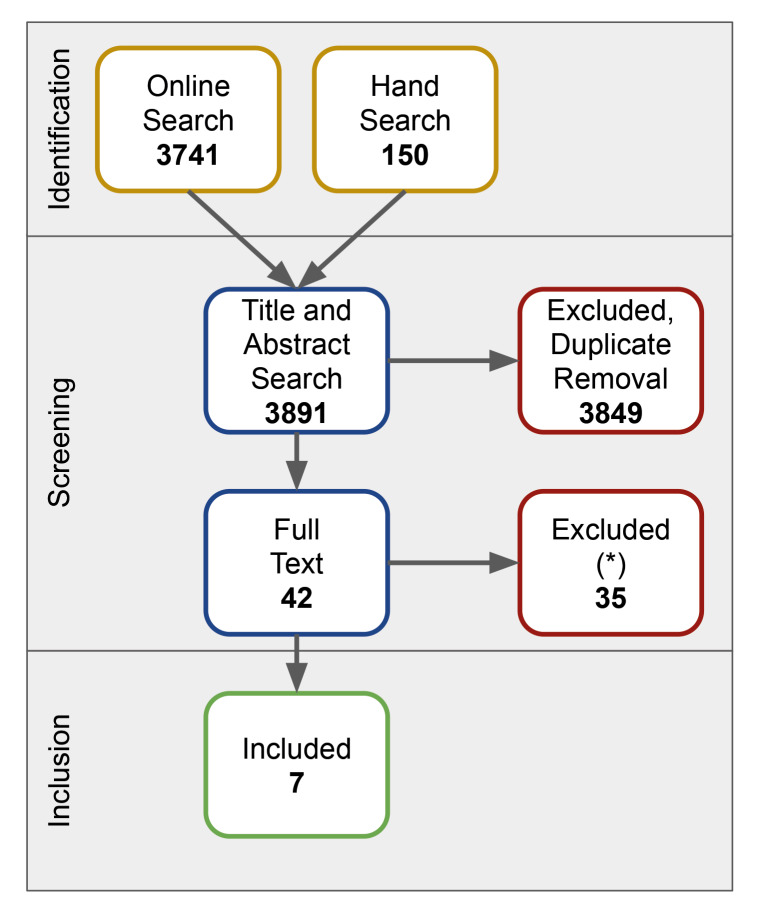
Prisma flowchart of the systematic search.

The electronic search, supplemented by a hand search, identified 3891 articles. Duplicate removal and title/abstract review led to an exclusion of 3849 articles. Forty-two (42) articles were considered for full-text screening. Thiry-five (35) articles were excluded at this stage. Justification for exclusion can be found in Supplemental Table S2.

Analysis of the level of agreement between reviewers demonstrated a high inter-rater reliability. Twenty (20) titles were chosen by chance to determine reliability and level of agreement, resulting in a 90% level of agreement.

### Risk of Bias Assessment (Table 1)

**Table 1:** Risk of bias assessment results of included studies

Study	Newcastle-Ottawa quality assessment scale	
Selection	Comparability	Exposure	
Is the case definition adequate? (Maximum: *)	Representativeness of the cases (Maximum: *)	Selection of Controls (Maximum: *)	Definition of Controls (Maximum: *)	Comparability of cases and controls based on the design or analysis (Maximum: * *)	Ascertainment of exposure (Maximum: *)	Same method of ascertainment for cases and controls (Maximum: *)	Non-Response rate (Maximum: *)	Total score (out of 9)
^Costa 2022^	*	*	*	*	**	*	*	*	*********(9)
^Costa 2014^	*	*	*	*	**	*	*	*	*********(9)
^König 2001^	*	*	*	*	**	*	*	*	*********(9)
^Kocher 2024^	*	–	*	*	**	*	*	*	********(8)
^Miyamoto 2006^	*	*	*	*	**	*	*	*	*********(9)
^Rieder 2004^	–	–	*	*	–	*	*	*	*****(5)
^Roccuzzo 2022^	*	*	*	*	**	*	*	*	*********(9)


The results of the risk-of-bias assessment can be found in Table 1. Of the seven included studies, five^4,5,11,17,22^ received a 9 points and 10 received 8 points, indicating a low risk of bias. One study^21^ received 5 points, implying a moderate risk of bias. This was primarily due to limitations in case definition, representativeness of the study population, and lack of group comparability due to design constraints.

### General Study Characteristics (Table 2)

**Table 2:** Characteristics of included studies

Study	Study Design	Observation [year]	Setting	Total Sample Size [n]	Study Populationaccording to study authors	Study Groups	Study Group Size [n]	Gender	Age [years, SD]	Follow up time at final exam [y, SD]		Number of visits between baseline and final [n, SD]	Number of visits/year [mean, SD]	Group Definition according to study authors	SPT interventionsaccording to study authors	resons for non attendanceaccording to study authors
a) SPT		
Costa F.O. et al. (2014)	ProspectiveCohort	5	Private practice	265 at baseline212 at final	patients undergoing periodontal maintenance program with previous diagnosis of chronic moderate-advanced periodontitis and at least 14 teeth	Reg	96	F: n=56 (58.7%)M: n=40 (41.3%)	≤40: 15y (15.7%)41-55y: 32 (33.3%) >55: 49 (51.0%)	61.2 months±3.4		11.2 ± 2.8	2.2 ± 0.55	100% adherent with maximum 6 months between SPT visits	• not reported	reasons not discussed
Irreg	116	F: n=52 (44.8%)M: n=64 (55.2%)	≤40: 21 (18.1%)41-55: 43 (37.1%) >55: 52 (44.8%)	62.6 months±3.9	5.1 ± 1.3	0.978 ± 0.249	missed SPT visits but continued to appear on irregular bases with maximal interval of 18 months between visits
König J.H.C. et al. (2001)	RetrospectiveCohort	10	University	230	moderate to advanced periodontitis patients in supportive perodontal therapy	reg	142	F: n=76M: n=66	46±9	10.5 +/- 1.6	not reported	not reported	adherent to SPT 10 years	• remotivation, reinstruction according to re-evaluated plaque index • recording of pocket probing depth at least once a yearsites with pocket probing depth >4 mm and/or bleeding on probing or suppuration received subgingival scaling without anesthesia • professional tooth-cleaning at every appointment	economic problems, socioeconomic status, fear, lack of satisfaction to behavior of the dentist
non-adh B	42	F: n=26M: n=16	48±9	3.0 +/- 2.6	discontinued SPT
non-adh C	44	F: n=26M: n=18	48±13	na	dropped out before or during surgery
Miyamoto T et al. (2006)	RetrospectiveCohort	15 to 23	Private practice	505	periodontitis patients with at least 10 years of supportive periodontal therapy, 6-month intervals	Complete 1	180	not reported	43.2±11.4	16.6 – 2.09	not reported	not reported	patients who missed < 30% of all prescribed SPT visits	• professional mechanical tooth cleaning, reinforcement of oral hygiene, review of updated medical history, evaluation of further change in the periodontium and tooth condition • If further treatment needed during maintenance: appropriate treatment provided, such as extraction of hopeless teeth, restorative treatment, endodontic therapy, and periodontal treatment	reasons not discussed
Complete 2	164	43.2±11.7	16.7 – 2.14	patients who never went 2 years without visit
Erratic 1	325	40.2±11.5	17.1 – 2.11	opposite of Complete 1
Erratic 2	341	40.3±11.4	17.1 – 2.09	opposite of Complete 2
Non-Adh	na	na	na	no show patients, never responded to SPT visits
Rieder H et al. (2004)	RetrospectiveCohort	7.3±1.5(minimum 5 years)	Private practice	87	periodontitis patients in supportive periodontal therapy	Fully Adh	24	F: n=52 (59.8%)M: n=35 (40.2%)	44.6+9.8	not reported	not reported	not reported	< 1 week delay from scheduled SPT visit	• Re-evaluation, recording of PPD, CoP, if site exhibited bleeding or PPD > 4mm: manual instrumentation, polishing of entire dentition, topical fluorides	reasons not discussed
Adh I	24	within 1 to 3 week delay from scheduled SPT visit
Adh II	20	within 3 to 6 week delay from scheduled SPT visit
Non-Adh	19	delay >6 weeks scheduled SPT visit
Kocher T et al. (2024)	RetrospectiveCohort	4	University	335	fully and partially adherent vs drop-out patients in supportive periodontal therapy	Fully and partially adherent	280	F: n=150 (53.6%)M: n=130 (46.4%)	56,5	5.5±4.5	not reported	not reported	includes fully and partially adh fully: at last one visit/yearpartially: average attendance but < 1/year	• oral hygiene re-instruction and re-motivation, annual examination of PD at six sites per tooth, subgingival debridement of residual pockets ≥5 mm, professional tooth cleaning and fluoridation	lack of motivation, private reasons or low social status with an associated lack of understanding
Drop-out	55	F: n=30 (55.4%)¿M: n=25 (44.6%)	56,8	8.3±3.8	did not attend further SPT
b) SPIC											
Costa F.O. et al (2022)	ProspectiveCohort	11	Private practice	80 as baseline51 at final exam	edentulous patients rehibliated with implants, diagnosed with periimplant mucositis participating in periimplant maintenance therapy (PIMT)	Reg	27	F: n=17 (62.9%)M: n=10 (37.1%)	59.2±10.8	not reported	not reported	not reported	100% adherence with a maximum of 9 months between visits	• periodontal and peri-implant status assessment, application of disclosing agents, oral hygiene instructions with use of conventional brushes, dental floss, interdental brush, encouragement to use oral irrigators, prophylaxis and non-surgical and surgical mechanical debridement, when necessary	several behavioral, social, cultural, and economic reasons , drastic and lasting changes related to a healthier life style difficult to maintain
Irreg	24	F: n=11 (45.9%) M: n=13 (54.1%)	62.5±10.3	missed visits but continued to appaear on an irregular basis with a maximum interval off 18 months between visits
Roccuzzo A et al. (2022)	ProspectiveCohort	20	Private practice	149 at baseline84 at final	tissue-level implants in partially edentulous patients previously treated for periodontitis and healthy patients in SPT	Adh PHP	17	not reported	not reported	not reported	not reported	not reported	S = number of pockets 5-7mm + 2*Number of pockets ≥8mma) Moderate PCP (mPCP): PCP with S ≤ 25b) Severe PCP (sPCP): PCP with S > 25adherence to SPC (yes or no): full compliance with an SPC program proposed by the principal investigator taking into account patient’s needs and risk profile	• oral hygiene instructions, biofilm removal and treatment of re-infected sites whenever needed, • diagnosis and treatment of peri-implant diseases according to concept of cumulative interceptive supportive therapy	reasons not discussed
Non-adh PHP	5
Adh mPCP	16
Non-adh mPCP	13
Adh sPCP	24
Non-adh sPCP	9
adh: adherence; (ir)reg: (ir)regular; PHP: periodontally healthy patients; PCP: periodontally compromised patients; m: moderate; s: severe; f: female; m: male; n: number; SD: standard deviation; y: year(s); na: not applicable/not reported

A total of seven articles published from 2001 to 2024 were deemed eligible for inclusion in this systematic review. Five of the seven^4,5,17,21,22^ were conducted in a private dental practice and two^10,11^ in a university clinic. All of the included studies were designed as cohort studies; three were prospective,^4,5,22^ and the remaining had a retrospective design.^10,11,17,21^ Sample sizes varied extensively across studies, from a minimum of 87 up to 335 total participants. The duration of observations varied as well, ranging from an average of 4 years to 20 years. Only two studies reported on peri-implant related parameters.^4,22^

#### Definition of Adherence and Study Groups

Adherence was defined individually within each study, resulting in substantial variability and occasional discrepancies across studies. Definitions of adherence ranged from regular attendance with no deviation from planned visits to more lenient interpretations allowing extended intervals between maintenance appointments. Conversely, non-adherence was variably defined, encompassing complete non-attendance, irregular attendance, or delayed compliance with scheduled visits. This inconsistency in operational definitions complicates comparability between studies. Furthermore, most studies did not clearly differentiate between maintenance intervals and actual adherence to these schedules, making it difficult to evaluate the specific impact of timing and frequency of visits on clinical outcomes.

Most included studies compared a group of adherent patients with one or more groups exhibiting non-adherence patterns.^4,5,10,11^ Rieder et al^21^ subdivided irregular attenders based on the delay in attending scheduled maintenance visits – for example, adherence I: 1 to 3 weeks delay, adherence II: 3 to 6 weeks delay, and non-adherence: more than 6 weeks delay. Miyamoto et al^17^ defined adherence in two distinct ways and created two separate subgroup classifications of “complete” and “erratic” compliers based on attendance consistency over different time frames. In contrast, Roccuzzo et al^22^ uniquely stratified participants based on their periodontal health status, creating three groups each for adherent and non-adherent patients: (i) periodontally healthy, (ii) moderately periodontally compromised, and (iii) severely periodontally compromised patients.

### Study Findings (Table 3)

**Table 3a:** Results of included studies: tooth-related outcomes

Study	Study Groups	PPD baseline [mm]	PPD final [mm: mean, SD]	PPD change	Tooth loss baseline [n]	Tooth loss final [n]		CAL baseline [mm]	CAL final [mm]	BoP baseline [%]	BoP final [%]	BoP change [%]	Plaque Index baseline [%]	Plaque Index final [%]	Plaque Index change [%]
a) SPT															
Costa F.O. et al. (2014)	Reg	≥ 4-5mm: 1.5±3.7≥ 6mm: 0.5±1.8	≥ 4-5mm: 3.5±4.1≥ 6mm: 0.9±0.3	na	3,8	4,4	≥ 4-5mm: 13.2±1.5 ≥6mm: 9.9±0.9	≥ 4-5mm: 12.9±1.5 ≥6mm: 8.1±1.3	24.6+ 4.2	24.9+ 5.1	na	33.4 ± 4.7	35.25 ± 5.23	na
Irreg	≥ 4-5mm: 1.7±3.5≥ 6mm: 0.7±1.7	≥ 4-5mm: 4.1±3.8≥ 6mm: 1.5±0.5	4,0	5,8	≥ 4-5mm: 13.4±1.5 ≥6mm: 10.2±1.5	≥ 4-5mm: 14.1±2.1 ≥6mm: 13.8±1.5	27.8+6.1	32.8+6.9	34.1 ± 5.9	44.98 ± 7.34
König J.H.C. et al. (2001)	reg	2.9±0.5	1y: 2.92Y: 3.03Y: 3.24Y: 3.2	na	na	na	na	na	na	na	na	na	na	na
non-adh B	3.1±0.6	1Y: 3.12Y: 3.73Y: 3.54Y: 3.8
non-adh C	na	na
Miyamoto T. et al. (2006)	Complete 1	% sites with < 3mm79.1 ± 21.3	Reduction in PPD OR 1.00	patients with improvement in % of sites PPD >3 mm58.3%		% of patients with tooth loss55.6%	na	na	38.1 ± 22.2	Reduction in BOP OR 1.00	% of patients with improvement66.7%	41.6 ± 21.1	Reduction in PI OR 1.00	% of patients with improvement71.7%
Complete 2	74.3 ± 24.4	OR 1.00	57,90%		51,2	41.6 ± 24..9	OR 1.00	60,40%	44.5 ± 23.1	OR 1.00	68,30%
Erratic 1	72.8 ± 24.1	OR 0.96 95% CI (0.65;1.40)	58,50%		40,3	42.6 ± 25.4	OR 0.78 95% CI (0.53;1.17)	63,40%	41.8 ± 23.2	OR 0.67 95% CI (0.44;1.01)	65,30%
Erratic 2	75.4 ± 22.9	OR 1.20 95% CI (0.81; 1.78)	58,70%		43,1	40.8 ± 24.2	OR 0.98 95% CI (0.67;1.44)	66,60%	44.3 ± 22.1	OR 0.90 95% CI (0.59;1.36)	67,30%
Non-Aadh	na	na	na		na	na	na	na	na	na	na
Rieder C. et al. (2004)	Fully Adh	% of residual pockets (PPD ≥ 5mm)4.6%	% of residual pockets (PPD ≥ 5mm)7.8%	3.2% ± 5.9	na	annual tooth loss0.11±0.14	na	na	na	na	na	na	na	na
Adh I	5,30%	7.9% ± 5.9	2.6% ± 4.1	0.14±0.20
Adh II	4,20%	7,60%	3.4% ± 9.0	0,11
Non-Adh	5,40%	11,20%	5.8% ± 16.1	0.17±0.26
Kocher T et al (2024)	Fully and partially adherent	2.68 ± 0.40	2.74±0.41	0.06±0.57	na	annual tooth loss0.19±0.55	na	na	na	% of sites with BOP 45.4±13.6	na	na	%of sites with Plague 42.9±23.9	na
Drop-out	2.76 ± 0.42	2.99±0.75	0.23±0.86	0.31±0.50	55.4±17.7	62.3±26.8
b) SPIC														
Costa F.O. et al. (2022)	Reg	% of sites with probing depths > 4mm4.8±8.7	2.3±4.8	na	3.8 ± 3.3	na	na	na	27.0 ± 19.6	25.7 ± 11.1	na	1.4 ± 0.7	1.5 ± 0.3	na
Irreg	8.2±12.8	4.8±7.4	5.9 ±4.5	37.4 ± 23.7	39.9 ± 13.9	1.9 ± 0.5	2.2 ± 0.6
Roccuzzo A et al. (2022)	Adh PHP	na	Deepest PD (mm)3.9 ± 0.9	na	na	teeth lost during SPT0.8 ± 0.3	na	na	na	21.0 ± 18.4	na	na	13.8 ± 17.0	na
Non-adh PHP	4.2 ± 2.6	0.8 ± 0.4	45.8 ± 29.3	33.3 ± 25.8
Adh mPCP	4.1 ± 1.4	1.0 ± 0.2	21.9 ± 26.3	20.5 ± 26
Non-adh mPCP	4.7 ± 1.5	0.9 ± 0.3	41.3 ± 27.8	41.3 ± 30.8
Adh sPCP	4.1 ± 1.3	1.0 ± 0.2	20.4 ± 22.1	11.7 ± 17.0
Non-adh sPCP	5.3 ± 1.0	1.1 ± 0.2	56.8 ± 20.7	63.8 ± 26.0
adh: adherence; (ir)reg: (ir)regular; PHP: periodontally healthy patients; PCP: periodontally compromised patients; m: moderate; s: severe; f: female; m: male; n: number; SD: standard deviation; y: year(s); PI: peri-implant; PD: probing depth; PPD: pocket probing depth; BoP: bleeding on probing; CAL: clinical attachment loss.

#### Periodontal Tissue Related Outcomes (Table 3a)

##### Pocket Probing Depth (PPD)

All included studies assessed PPD as a clinical parameter, although the reporting formats varied. Some studies presented mean values with standard deviations, while others reported the number or percentage of sites exceeding specific thresholds (e.g. ≥4 mm or ≥6 mm).

In general, most studies – except Miyamoto et al^17^ and Rieder et al^21^ – found statistically significant reductions in PPD among regular attenders compared to irregular attenders. Notably, Roccuzzo et al^22^ reported this difference only in the subgroup of severely periodontally compromised patients.

Miyamoto et al^17^ and Rieder et al^21^ did not observe any statistically significant differences in PPD between adherence groups.

Importantly, Kocher et al^10^ conducted two separate analyses: one including and one excluding data from extracted teeth. When extracted sites were excluded, mean PPD was statistically significantly lower in regular attenders. When measures around extracted teeth were included, mean PPD did not differ statistically significantly between groups; however, the percentage of sites with PPD ≥4 mm and ≥6 mm remained statistically significantly higher in irregular attenders.

##### Other Outcomes

Five included studies investigated tooth loss rates. The results were heterogeneous: Three studies found statistically significantly higher tooth loss rates in non-adherent patients compared to regular attenders (p < 0.05). In Roccuzzo et al,^22^ however, this difference was statistically significant only in the subgroup of severely periodontally compromised patients.^5,10,22^ Kocher et al^10^ calculated an incidence rate ratio of 2.20 (95% CI: 1.31– 3.70). Rieder et al^21^ reported no statistically significant difference between the study groups regarding tooth loss rates. Interestingly, Miyamoto et al^17^ observed higher tooth loss rates in adherent patients compared to erratic attenders, but this was only observed under one of their two definitions of adherence.

Four out of five studies reporting on bleeding on probing and plaque index scores reported reduced BoPs in regular attenders compared to non-adherent patient groups.^4,5,10,22^ Miyamoto et al^17^ reported no statistically significant differences between groups.

Differences in clinical attachment loss were reported in only one study^5^ and showed statistically significantly higher rates in the non-adherent group (p < 0.05).

#### Peri-implant Tissue Related Outcomes (Table 3b)

**Table 3b:** Results of included studies: peri-implant outcomes

Study	Study Groups	PI Diagnosis baselinepatient level	PI Diagnosis final patient level	PI BoP % baseline	PI BoP % final		PI-PD baseline [mm]	PI-PD final [mm]	PI bone loss baseline	PI bone loss final	Implants with periimplantitis baseline [n]	Implants with periimplantitis final [n]	PI keratinised mucosa baseline	PI keratinised mucosa final
SPIC														
Costa F.O. et al. (2022)	Reg	Healthy: 12 (44.4%)peri-implant mucositis: 20 (74.1%)peri-implantitis: 7 (25.9%)	Healthy: 17 (63.0%)peri-implant mucositis: 10 (37.0%)peri-implantitis: 4 (14.8%)	33.3 +/- 25	24.2 +/- 14.2	% of sites with PI PD > 4mm5.9 +/- 13.6	3.3 +/- 8.4	individuals with bone lossn=7	n=4	17 (10.6%)	6 (4.8%)	% of sites with KMi ≤1 mm9.0 ± 18.5	18.9 ± 21.2
Irreg	Healthy: 0 (0.0%)peri-implant mucositis: 23 (95.8%)peri-implantitis: 18 (75.0%)	Healthy: 6 (25.0%)peri-implant mucositis: 17 (70.8%)peri-implantitis: 10 (41.6%)	62.7 +/- 20.9	39.3 +/- 19.8	16.7 +/- 27.0	10.1 +/- 13.2	n=17	n=10	52 (28.8%)	24 (22%)	7.7 ± 15.9	21.9 ± 22.7
Roccuzzo A et al. (2022)	Adh PHP	patients treated with CIST C/D (10 y)4 (23.5%)	patients treated with CIST C/D (20 y)5 (29.4%)	na	na	at least 1 site with deepest PD ≥6 mm at 10 years3 (17.7%)	at least 1 site with deepest PD ≥6 mm at 20 years4 (23.5%)	na	na	na	na	na	na
Non-adh PHP	1 (20%)	2 (40%)	2 (40%)	4 (80%)
Adh mPCP	9 (52.9%)	6 (35.3%)	5 (29.4%)	6 (35.3%)
Non-adh mPCP	4 (30.8%)	8 (61.5%)	11 (84.6%)	10 (76.9%)
Adh sPCP	15 (62.5%)	16 (66.7%)	16 (66.7%)	10 (41.7%)
Non-adh sPCP	4 (44.4%)	3 (33.3%)	9 (100%)	9 (100%)
adh: adherence; (ir)reg: (ir)regular; PHP: periodontally healthy patients; PCP: periodontally compromised patients; m: moderate; s: severe; f: female; m: male; n: number; SD: standard deviation; y: year(s); PI: peri-implant; PD: probing depth; PPD: pocket probing depth; BoP: bleeding on probing; CAL: clinical attachment loss.

##### Peri-implant probing depth (PI-PD)

Only two studies reported outcomes specifically related to peri-implant tissue health during the supportive maintenance phase.^4,22^

Both studies demonstrated increased PI-PD in non-regular attender study groups. While the findings by Costa et al^4,5^ demonstrated that the percentage of sites exhibiting increased probing depths (>4 mm) decreased in both the regular and irregular attender groups, the overall percentage was markedly lower in the regular attender group both at baseline and at the final included measurement timepoint (regular baseline 5.9 ± 13.6, final: 3.3 ± 8.4, irregular baseline 16.7 ± 27.0, final: 10.1 ± 13.2, p < 0.001). Roccuzzo et al,^22^ who sub-grouped the population based on their periodontal health, showed statistically significant differences of PI-PD, measured as percentage of sites with deeper PI-PD ≥ 6 mm, both at 10-year and 20-year observation timepoints in each of these subgroups (periodontal health at 20 years: non-adherent 80%, adherent: 23.5%, p < 0.04; moderate periodontally compromised patients at 10 years: non-adherent 84.6%, adherent 29.4%, p < 0.02; severely periodontally compromised patients at 20 years: non-adherent 100%, adherent 41.7%, p < 0.04). These findings highlight not only the influence of adherence on peri-implant tissue health but also underscore the importance of stratifying patients by periodontal status when assessing long-term peri-implant stability.

##### Additional Outcomes

Both studies also reported on peri-implant bone loss and peri-implantitis occurrence. In the study by Costa et al,^4^ bone loss (p < 0.002) and the diagnosis of peri-implantitis at both the patient and implant level (p < 0.05) were statistically significantly more frequent in the irregular attender group compared to regular attenders. Additionally, statistically significantly lower peri-implant bleeding scores were observed in the regular attender group (p < 0.012).

Roccuzzo et al^22^ evaluated the proportion of patients treated according to the Cumulative Interceptive Supportive Therapy (CIST, 12 ) protocols C (pocket formation/increased PI-PD) and D (progressive bone loss, suppuration, deep pockets) at 10- and 20-year follow-ups. However, no statistically significant differences between regular and irregular attenders were found within these subgroups. It should be noted that Roccuzzo et al^22^ assessed adherence based on therapy delivered (CIST categories), while Costa et al^4^ focused on direct clinical parameters, which limits comparability between the two studies.

## DISCUSSION 

Our aim was to assess whether clinical outcomes associated with adherence or non-adherence to SPC/SPIC relate to the maintenance of periodontal or peri-implant stability. While the 2018 classification defines periodontal and peri-implant health through specific clinical thresholds, the term “stability” is used here descriptively to reflect the maintenance of favorable clinical status over time. While the included studies showed consistent trends favoring better outcomes in adherent patients, the heterogeneity in adherence definitions, measurement protocols, and outcome parameters limited our ability to quantitatively estimate the magnitude of this effect. Nevertheless, the present findings underscore the clinical relevance of adherence, particularly in maintaining periodontal and peri-implant tissue stability over time.

Across the studies included, improvements in probing depths, bleeding scores, and tooth/implant survival were generally associated with regular attendance at maintenance visits. However, this association must be interpreted with caution, as reasons for non- or irregular adherence were either inconsistently reported or not discussed at all. When provided, these reasons ranged from socioeconomic hardship, lack of motivation, and dissatisfaction with dental care to psychological and lifestyle-related factors. For example, König et al^11^ and Costa et al^4^ alluded to personal and financial challenges, while also highlighting the difficulty of sustaining major lifestyle changes over time. Kocher et al^10^ also mentioned lack of motivation as a reason for poor adherence, while also highlighting that lack of understanding may have an influence.

These findings align with existing literature that has identified multiple barriers to adherence. Helal et al^8^ reported that low health literacy, insufficient patient education, and unclear communication were key predictors of non-compliance. Similarly, Wilson^27^ emphasized that behavioral and cultural components, as well as dissatisfaction with the dentist, elevated the risk of non-attendance. These data suggest that effective SPC/SPIC programs must go beyond clinical scheduling and actively address behavioral, educational, and systemic factors that influence patient commitment.

Despite the clinical relevance of the findings, the heterogeneity across studies precluded a formal meta-analysis. Adherence was defined inconsistently, with varying thresholds, recall intervals, and behavioral categorizations across studies. Additionally, the protocols for supportive care were often insufficiently described, and the reporting of outcomes lacked standardization, ranging from mean probing depths to proportions of affected sites or categorical outcomes like tooth loss. Importantly, none of the studies stratified participants by initial periodontitis grade or stage, which critically affects treatment response and recall interval justification. These factors, in combination with variable follow-up durations, make quantitative synthesis inappropriate. We have, therefore, opted for a narrative synthesis to preserve the integrity and interpretability of the included data.

We acknowledge that a one-year follow-up may not fully capture long-term periodontal or peri-implant stability, given the chronic and episodic nature of these diseases. However, this threshold was selected as a pragmatic inclusion criterion to ensure that all patients had received at least one complete cycle of supportive care. Furthermore, this duration is consistent with other reviews in the field and reflects the minimum interval in which clinically relevant changes may emerge under maintenance protocols. Nonetheless, all included studies substantially exceeded this minimum, and the present authors interpreted findings with respect to their specific follow-up durations throughout this work. Future reviews may benefit from stratifying outcomes by follow-up length to better reflect time-dependent effects of adherence.

### Primary Outcome Measure

In this review, PPD/PI-PD was chosen as the primary clinical outcome measure to evaluate periodontal and peri-implant stability. While newer frameworks propose more comprehensive definitions of periodontal health, PPD/PI-PD remains a well-established and widely recorded clinical parameter in both research and routine practice as is recognised as a conventional, sensitive, and essential measure in periodontal practice.^6^ Although the sensitivity is lower, this measure is also widely accepted in peri-implant disease monitoring.^18^ According to current guidelines and supporting literature,^15^ PPD demonstrates sufficient prognostic utility, particularly in its association with tooth loss risk. Moreover, although clinical attachment level (CAL) may be considered a more definitive indicator of tissue support, it is often less reliably recorded in routine settings. In contrast, PPD measurements are consistently documented across studies, enhancing comparability. As such, the use of PPD/PI-PD as a primary outcome in this context provides a practical and clinically meaningful metric for assessing long-term treatment success.

There are a few published systematic reviews investigating adherence (previously compliance) to supportive periodontal and peri-implant care, but the main outcomes are focused around tooth loss/tooth retention.^1,2,13^

### Limitations

Several limitations were encountered during the preparation of this systematic review.

Firstly, only a limited number of studies were identified that directly addressed the research question according to our predefined PECOS criteria. While all included studies compared adherence to non-adherence in supportive periodontal or peri-implant care, the definitions of adherence varied substantially between studies. Some studies considered adherence as strict compliance to recommended intervals, while others allowed for broader interpretations, including partial or delayed attendance. This variability hinders comparability and precludes quantitative synthesis such as meta-analysis. This limitation and lack of clear definition on adherence and a clear consensus on this has been highlighted in previous studies as well.^3^

Secondly, none of the included studies stratified participants according to the initial stage or grade of periodontitis, despite this being a core determinant of disease progression and treatment response. The absence of such stratification is particularly limiting, as recent evidence has shown that patients with more advanced periodontal disease exhibit more pronounced negative outcomes when adherence is lacking.^22^

Furthermore, the scarcity of data on supportive peri-implant care (SPIC) remains a major gap. Only two studies reported peri-implant-related outcomes, and these differed markedly in their methodologies and reporting standards. There was also considerable heterogeneity in clinical measurement protocols, such as probing depth thresholds, time points of measurement, or whether extracted teeth/implants were included in final analyses.

Another common shortcoming was the inconsistent or absent reporting of key confounding factors, such as smoking status or systemic health conditions (e.g., diabetes mellitus). These are well-established modifiers of periodontal and peri-implant outcomes and their omission may lead to biased or incomplete interpretations of adherence effects. Despite these limitations, the available data collectively support the clinical relevance of adherence to SPT/SPIC, underscoring the need for standardized reporting, stratification by baseline risk, and better control for systemic and behavioral confounders in future research.

### Comparison to Existing Literature

Previous systematic reviews have explored the long-term effectiveness of SPC and SPIC, but have done so with different focal points. Leow et al^14^ investigated recurrence and progression of periodontitis during SPC, identifying a generally low rate of tooth loss over time. However, the review did not consider adherence as a variable, and instead broadly described outcomes in populations assumed to be receiving SPC. Similarly, Carra et al^3^ reviewed prevention strategies for peri-implant diseases, including comparing patients receiving vs not receiving SPC/SPIC. Yet again, adherence was neither consistently defined nor treated as a structured exposure.

Other systematic reviews exist which take a closer look at adherence (previously: compliance) in the population of periodontitis patients in supportive therapy. Amerio et al^1^ included 19 studies identifying predictors of non-compliance (now: non-adherence). Although they commented on adherence rates and were able to clarify that the most common reason for non-compliance is the lack of understanding and or motivation of the patient, clinical outcomes were not analyzed.

According to a systematic review by Manresa et al,^16^ non-adherence to SPT leads to an increased risk of tooth loss. Despite investigating a fairly similar hypothesis, their main outcome of tooth loss is an absolute finding, which can be related to a variety of factors beyond periodontitis (e.g., decay, fractures). Further, implant patients were not been included, nor was SPIC investigated.

In contrast, the present review specifically focused on adherence to SPC/SPIC and its influence on clinical parameters of stability. Across all included studies, higher levels of adherence were consistently associated with better outcomes, such as reduced probing depth, lower bleeding on probing, and fewer instances of tooth or implant loss. Importantly, we highlight the variability in how adherence is defined and reported, which has not been systematically addressed in previous reviews. By critically appraising this heterogeneity, our findings provide a more targeted understanding of how adherence patterns influence long-term stability, offering practical implications for both research and clinical practice.

## CONCLUSION

This systematic review demonstrates that adherence to supportive periodontal (SPT) and peri-implant care (SPIC) positively influences the long-term maintenance of periodontal and peri-implant stability. Across all included studies, patients who adhered to maintenance protocols exhibited more favorable clinical outcomes, such as reduced probing depths, less bleeding on probing, and lower rates of tooth or peri-implantitis, compared to non-adherent individuals.

Despite the consistency in this trend, heterogeneity in the definitions of adherence and clinical outcome measures across studies limited direct comparability and precluded meta-analysis. Additionally, most studies lacked detailed reporting on how systemic, behavioral, or environmental confounding factors were considered in assessing outcomes. Nevertheless, the findings emphasize the need for regular, individualized SPT/SPIC protocols that are based on measurable clinical parameters and supported by thorough documentation and reassessment during follow-up visits.

Importantly, future studies and clinical practice should incorporate risk-based stratification according to the initial periodontitis staging and grading, as patients with higher disease severity may experience more adverse outcomes if maintenance care is suboptimal. This was clearly illustrated in the findings of Roccuzzo et al,^22^ where statistically significantly worse peri-implant outcomes were observed in non-adherent patients with severe periodontitis. A clear and consistent structure for recording and implementing maintenance intervals is therefore essential, as patients with low-stage, low-grade periodontitis may not require the same frequency or intensity of maintenance as those with high-stage, high-grade disease.

The current literature remains limited particularly regarding peri-implant health, and future studies should apply standardized definitions of adherence, account for confounding risk factors, and adopt consistent data collection methodologies. Also, patient-reported outcomes should be added. More evidence is also needed to guide SPIC in patients with dental implants, where definitions, intervals, and outcomes remain inconsistently reported.

This review underscores the critical importance of adherence to maintenance care in preserving oral health and achieving long-term treatment success.

### Clinical Relevance

Adherence to supportive periodontal therapy (SPT) and supportive peri-implant care (SPIC) is crucial for maintaining oral health and preventing disease recurrence. Regular attendance at maintenance appointments has been associated with reduced tooth and implant loss, decreased financial burden, and minimized discomfort during the maintenance period. Conversely, non-adherence can lead to further loss of tooth and implant-supporting tissues and higher overall treatment costs. Emphasizing the importance of adherence during patient education and addressing individual risk factors can enhance motivation and compliance. Implementing evidence-based strategies to improve adherence can lead to better clinical outcomes and more efficient resource utilization in dental practice.

## REFERENCES 

## Supplemental Tables

**Table S1** Electronic search terms

**Table:** 


(“periodont*”[All Fields] OR “peri implant*”[All Fields] OR “periimplant*”[All Fields]) AND (“compliances”[All Fields] OR “patient compliance”[MeSH Terms] OR (“patient”[All Fields] AND “compliance”[All Fields]) OR “patient compliance”[All Fields] OR “compliance”[All Fields] OR “compliance”[MeSH Terms] OR (“compliants”[All Fields] OR “patient compliance”[MeSH Terms] OR (“patient”[All Fields] AND “compliance”[All Fields]) OR “patient compliance”[All Fields] OR “compliant”[All Fields]) OR (“adherance”[All Fields] OR “adhere”[All Fields] OR “adhered”[All Fields] OR “adherence”[All Fields] OR “adherences”[All Fields] OR “adherent”[All Fields] OR “adherents”[All Fields] OR “adherer”[All Fields] OR “adherers”[All Fields] OR “adheres”[All Fields] OR “adhering”[All Fields]) OR “SPT”[All Fields]).


**Table S2** List of excluded studies, including justification in full-text search stage

**Table:** 

Reasons for exclusion	First author, year
Data not reported according to our PICO (n=15)	Soolari 2003, De Tapia 2022, Hägi 2013, Roccuzzo 2023, Jönsson 2006, Wilson 1986, Pretzl 2018, Chambrone 2006, Bartha 2022, Bäumer 2018, van der Moolen 2020, Checci 2002, Aquirre-Zorzano 2014, Petit 2019, Kawahara 2020
Study groups not according to our PICO (n=16)	Kakudate 2010, Ramseier 2014, Ramseier 2018, Graetz 2017, Costa 2006, Costa 2012, Lorentz 2009, Aquirre-Zorzano 2014, Angst 2019, König 2002, Müller-Campanille 2019, Schoemakers 2022, Frisch 2014, Frisch 2020, Ravidà 2021, Sonnenschein 2020
Book articles (n=1)	Wilson 1990
Same cohort (n=1)	Miyamoto 2010
Article not available (n=2)	Svenson 1989, Fardal 2003


**Table S3** PRISMA Checklist




